# Then and now: lessons learned from community- academic partnerships in environmental health research

**DOI:** 10.1186/s12940-016-0201-5

**Published:** 2016-11-29

**Authors:** Maureen Lichtveld, Bernard Goldstein, Lynn Grattan, Christopher Mundorf

**Affiliations:** 1 Department of Global Environmental Health Sciences, Tulane University School of Public Health and Tropical Medicine, New Orleans, LA USA; 2 Department of Environment and Occupational Health, University of Pittsburgh, Pittsburgh, PA USA; 3 University of Maryland School of Medicine, Baltimore, MD USA; 4 Hiram College, Hiram, OH USA

**Keywords:** NIEHS, Community-academic partnerships, Community-based environmental health research, Citizen science

## Abstract

On the occasion of the 50^th^ anniversary of the National Institutes of Environmental Health Sciences we reflect on how environmental research incorporating community members as active partners has evolved, benefited communities and advanced environmental health research. We highlight the commitment to community partnerships in the aftermath of the 2010 Deep Water Horizon Oil Spill, and how that commitment helped improve science. We provide examples of community-academic partnerships across the engagement spectrum. Finally, we offer suggestions to improve the community engagement in order to cultivate more long partnerships and better scientific research.

## Background

The 2010 Deepwater Horizon (DWH) Oil Spill resulted in extraordinary visibility and pressure to address the ecosystem impact of this unprecedented disaster. Despite the largest ever occupational epidemiologic study involving 32,000 workers undertaken by NIEHS internally [1], a massive outcry to address the health consequences in Gulf Coast communities persisted. Communities not only presented specific concerns but strongly urged that those concerns be examined with them as partners, rather than as traditional study subjects [2]. This commentary highlights examples of how these community-academic partnerships advanced environmental health research and examines lessons learned in the context of the DWH oil spill.

### Brief history of NIEHS involvement in community-based research activities

The National Institutes of Health (NIH) postwar expansion was based upon recognition of the value of peer-reviewed research predominantly by physician-scientists who effortlessly could move from laboratory to clinic and back again. The NIH grants program grew from $4 million in 1947 to $100 million in 1957 and to $1 billion in 1974 accompanied by major growth in its intramural program [3]. New institutes were developed based upon traditional divisions among medical specialties and subspecialties or upon organ groupings and disease types. But NIEHS was conceived differently.

Initially, the critical health impact of NIEHS-funded research occurred through translation and application to national policies. Responding to national concerns about air and water pollution, and toxic agents such as lead, poly chlorinated biphenyls and benzene, these research endeavors led to policies that saved many lives. NIEHS research also generated the tools to prevent introduction of new chemicals with undesirable properties such as mutagenesis and neurotoxicity. Unfortunately, with NIH as a whole focusing on the environmental impacts on individual patients, and NIEHS focusing on basic science research leading to changes in public policy, communities received little attention. However, over time there was a growing appreciation for striking differences among communities in environmental hazards, perceived risks, and behavioral reactivity that were central to understanding and ameliorating environmental health impacts. Recognizing the importance of community-based research, the leadership of NIEHS, −with special credit to Directors Ken Olden and Linda Birnbaum-, advocated this as a core area of environmental health science, including mandating that all NIEHS-funded Research Centers have an outreach program focusing on the community. Additional NIEHS-led research advocated for greater emphasis on community based participatory research [4].

### Community collaboration in disaster-related environmental health research

The NIEHS commitment to community partnerships is often tested in response to natural and man-made disasters [3, 4]. Concerns about public health risks compounded by the vast ecosystem damage resulting from DHW oil spill and widespread regional distrust fueled community fears of poor environmental quality, neglect, and perceived forms of environmental racism [5]. In addition to a large intra-murally implemented worker study [6], the NIEHS-funded DWH Disaster Research Consortia were established as four regional academic-community partnerships to address Gulf coast residents’ health concerns, enhance capacity to respond to potential future disasters, and minimize potential adverse health effects [7]. The research consortia were guided by two key requirements aimed at fostering community engagement: targeted efforts across consortia towards developing -specific innovative approaches to better incorporate the community into the research process post oil spill, and at least one research project dedicated to explore community resilience. To assure community engagement, a specific budget line stipulated the creation of a Community Outreach and Dissemination Core (CODC), funding 39 community partners to actively engage in the proposed research. This engagement spanned the continuum from involvement in agenda setting through Community Advisory Committees and frequent progress meetings, to participating as full and funded research partners in specific research projects. NIEHS spearheaded the development and implementation of a CODC working group representing all 39 CBOs and charged with developing common consortia-wide products aimed at disseminating joint research findings to all communities. CODC partners are now actively engaged in the research dissemination phase- both in overarching activities (e.g. joint factsheets) and project-specific actions such as community meetings, and media networking [8]. The working group played a critical role in collaborating with academic partners to avoid conflicting messages while preserving scientific rigor. These efforts are the building blocks for a broader agenda on advancing environmental health literacy supporting NIEHS budding focus on a more evidence-based approach in this area [9].

### Advancements in chemical stressor research

At the project level, significant advances in environmental health research were facilitated through innovative community collaborative techniques: for example, researchers worked with community partners in planning, and implementing a targeted health risk assessment of seafood most relevant to a local Vietnamese community [10]. Mary Queen of Vietnam (MQVN), Tulane University’s transdisciplinary research consortium on Gulf Resilience On Women’s Health (GROWH) community partner, provided critical leadership in seafood collection so that the subsequent environmental analysis indeed could answer the question “is the seafood safe to eat” in a relevant and trustful fashion [10, 11]. Locally relevant data were used to inform risk assessment and management actions. The demographic assessment indicated residents in the Vietnamese community in New Orleans East had lower average body weights and consumed higher levels of seafood than the standard levels used in the traditional risk assessment equation. Specifically, the shrimp consumption rate of the partner community was more than triple that of NHANES 90^th^ percentile rate [9]. Despite this revision, the researchers documented that the seafood was safe to eat. This approach allowed for more buy-in from the community, and also provided invaluable input into the cultural factors that could modify risk. Furthermore, informed by the research priorities delineated by the Institute of Medicine [12], consortia investigators established a Seafood Exposure Assessment Working Group delineating best practices for seafood collection and analysis. [13]. This method resulted in a coherent strategy to address the seafood safety community concern, promoting effective research translation and avoiding community confusion [14]. Specifically, the CODC used the results to develop a factsheet posted on the NIEHS website [15].

Similar to MQVN, Bayou Interfaith Shared Community Organizing (BISCO), another GROWH community partner, was actively engaged in the placement and collection of paired indoor and outdoor air monitors [16]. The analysis revealed preventable indoor air exposures that are now being addressed with active community involvement. The contaminants measured indoor (e.g. chloroform) were more likely linked to household cleaning product). At their request, the participants elected to receive their results via email with follow-up options through one on one consultation either in-home or by telephone as preferred. Furthermore, collaboration with the parish (county) President resulted in broader community dissemination of ways to improve indoor air quality. Accordingly, community partners are co-authors of publications highlighting study results [8, 17, 18]. Building on the success of these participatory engagements, MQVN, BISCO, the Louisiana Public Health Institute, and Tulane have developed a collaborative Community- Based Participatory Research Curriculum as a global model to strengthen community engagement in environmental health research. The 8- module curriculum is accompanied by lesson plans and an evaluation logic model assessing performance at the learner and program level [19]. Community members, all represented in Tulane University’s GROWH consortium’s CODC, co-developed each of the models as well as a logic model Under girding the evaluation framework [20].

“*Building Resilience Through Disaster Mobile Health”* is illustrative of the most intensive community engagement at the project level: community members were trained and embedded in the study design through the community health worker (CHW) model [17]. Data showed that the therapeutic relationship between the CHW and prenatal, health disparate women was associated with the participant’s psychosocial health, and independently predicted their study adherence in the longitudinal intervention. This relationship was also predictive of anxiety and depression symptoms post-partum [17, 21].

### Advancements in nonchemical stressor research

Research documented the combined effects of the oil spill with the other disasters (most notably Hurricane Katrina) that have affected the South-East Louisiana communities in recent years [22, 23]. The DHW oil spill produced widespread concern over lasting impacts on community health [12]. Several studies focused on mental health distress in vulnerable populations such as women [24] and children [25], and showed oil spill exposure (either directly or indirectly) was strongly related to poor behavioral health [2]. Working with communities in conflict, Mayer et al.[26], discovered that the operations of British Petroleum’s compensation program introduced a significant stressor, above and beyond the initial trauma of the oil spill. Colten et al. [27] documented ways culturally- inherent resilience pracices maximize post-oil spill functioning in several Louisiana communities. Gulf Coast community leaders worried about the mental health of their residents, seeking information regarding best coping strategies to manage distress. NIEHS-funded studies found that *active coping strategies* buffer negative mental health outcomes during the oil spill disaster, while the increased use of *disengagement coping* was associated with higher levels of anxiety and depression both during [24] and up to five years (linear regression model, *p* < .001) thereafter. With respect to the burden of intensive media coverage, one year after the DWH oil spill, the total time an individual was re-exposed to the spill and its environmental consequences through television, radio, print media and internet sources was not associated with overall mental health, but was significantly associated with hypervigilance, one symptom of anxiety [28]. These findings as well as the knowledge that environmental worry, persistent anger, and social support deterioration continued to predict poor mental health outcome over time were incorporated into community outreach, social, and psychological intervention programs through ongoing communication and engagement with area social service, and educational programs and agencies.

Coalescing multiple areas of scientific and public health expertise, the DHW research consortia developed a Resilience Activation Framework (RAF) to model the multidimensional nature of resilience. Incorporating individual, community, and cultural parameters, the central premises of the model were that 1) most people and communities have the inherent capacity to be resilient and 2) these latent capacities can be activated through social support [29]. Data obtained to date from the RAF are being used to increase access to social support networks through social, religious, educational, and occupational groups at the local level. However, challenges remain regarding the specific individual mechanisms needed to successfully “activate” and benefit from these networks.

## Conclusions

From design to dissemination, the benefits of engaging community partners are wide- ranging: from assuring research projects target practical and relevant research questions and innovative answers, to improving environmental health risk assessment, management and communication practices by generating locally relevant data, implementing community-driven interventions, and disseminating culturally- tailored information [17, 30, 31]. However, much remains to be done. Facilitated by the CODCs, communities engaged with each of the consortia shared their key questions that could be answered by leveraging existing data as well as exploring new funding opportunities. This includes research investments targeting early life exposures, preserving valuable cohorts, and allowing for expanded analysis of banked biospecimens, as well as transdisciplinary long-term environmental disaster outcome studies integrating chemical and psychosocial stressors. NIEHS leadership support of community-based research cannot be taken for granted. It depends upon our collective ability to provide impactful answers to community environmental problems.

## Abbreviations

BISCO: Bayou Interfaith Shared Community Organizing
CBO: Community-based organizations
CHW: Community health worker
CODC: Community outreach and dissemination core
DWH: Deepwater horizon
GROWH: Gulf resilience on women’s health
MQVN: Mary Queen of Vietnam
NHANES: National health and nutrition examination survey
NIEHS: National Institutes of Environmental Health Sciences
NIH: National Institutes of Health
